# Inter-epidemic Acquisition of Rift Valley Fever Virus in Humans in Tanzania

**DOI:** 10.1371/journal.pntd.0003536

**Published:** 2015-02-27

**Authors:** Robert David Sumaye, Emmanuel Nji Abatih, Etienne Thiry, Mbaraka Amuri, Dirk Berkvens, Eveline Geubbels

**Affiliations:** 1 Ifakara Health Institute, Ifakara, Tanzania; 2 Department of Biomedical Sciences, Institute of Tropical Medicine, Antwerp, Belgium; 3 Department of Infectious and Parasitic Diseases, Faculty of Veterinary Medicine, University of Liege, Liege, Belgium; The Kenya Medical Research Institute, KENYA

## Abstract

**Background:**

In East Africa, epidemics of Rift Valley fever (RVF) occur in cycles of 5–15 years following unusually high rainfall. RVF transmission during inter-epidemic periods (IEP) generally passes undetected in absence of surveillance in mammalian hosts and vectors. We studied IEP transmission of RVF and evaluated the demographic, behavioural, occupational and spatial determinants of past RVF infection.

**Methodology:**

Between March and August 2012 we collected blood samples, and administered a risk factor questionnaire among 606 inhabitants of 6 villages in the seasonally inundated Kilombero Valley, Tanzania. ELISA tests were used to detect RVFV IgM and IgG antibodies in serum samples. Risk factors were examined by mixed effects logistic regression.

**Findings:**

RVF virus IgM antibodies, indicating recent RVFV acquisition, were detected in 16 participants, representing 2.6% overall and in 22.5% of inhibition ELISA positives (n = 71). Four of 16 (25.0%) IgM positives and 11/71 (15.5%) of individuals with inhibition ELISA sero-positivity reported they had had no previous contact with host animals. Sero-positivity on inhibition ELISA was 11.7% (95% CI 9.2–14.5) and risk was elevated with age (odds ratio (OR) 1.03 per year; 95% CI 1.01–1.04), among milkers (OR 2.19; 95% CI 1.23–3.91), and individuals eating raw meat (OR 4.17; 95% CI 1.18–14.66). Households keeping livestock had a higher probability of having members with evidence of past infection (OR = 3.04, 95% CI = 1.42–6.48) than those that do not keep livestock.

**Conclusion:**

There is inter-epidemic acquisition of RVFV in Kilombero Valley inhabitants. In the wake of declining malaria incidence, these findings underscore the need for clinicians to consider RVF in the differential diagnosis for febrile illnesses. Several types of direct contact with livestock are important risk factors for past infection with RVFV in this study’s population. However, at least part of RVFV transmission appears to have occurred through bites of infected mosquitoes.

## Author Summary

Rift Valley fever (RVF) is a disease of animals and people that is caused by the RVF virus. During epidemics, humans get RVF through direct contact with animals or through mosquito bites. In East Africa, epidemics occur every 5–15 years following unusually high rainfall. In between epidemics, the transmission of RVF might occur at low level. In an epidemic-free period, we measured whether people in the Kilombero Valley in Tanzania had evidence of past and recent RVF infection in their blood sample, and studied risk factors. Three per cent of people had been infected recently, and 12% had evidence of past infection, with increased risk with age, among milkers and among people eating raw meat. Some people with past or recent infection reported they had not had contact with animals. Households keeping livestock had more members with evidence of past infection. The findings show that people get infected with RVF in between epidemics, and that various types of contact with livestock are important risk factors. There is also evidence that some people get infected with RVFV by mosquitoes in the epidemic free period. Clinicians in the Kilombero Valley should consider RVF in the differential diagnosis of patients with fever.

## Introduction

Rift Valley fever (RVF) is one of the major viral zoonoses in Africa. The disease is caused by the Rift Valley fever virus (RVFV) of the genus *Phlebovirus* in the family *Bunyaviridae* [1], and it is transmitted to animals through infectious mosquito bites and other arthropod vectors [2]. People become infected either from mosquito bites or by direct or indirect contact with infectious material when exposed to blood, body fluids or tissues of viraemic animals when handling sick or dead animals as well as through aerosol transmission, consumption of raw milk, meat or blood [3–5].

The disease was first described in the Rift Valley of Kenya in the early 1900s and the etiological agent demonstrated in the early 1930s [6]. RVF epidemics occur in cycles of 5–15 years in the Eastern Africa region as a result of abnormally high precipitation, for example during the warm phase of the El Niño/Southern Oscillation (ENSO) phenomenon [7]. In other regions the disease has been driven by floods caused by other sources including construction of hydroelectric dams [8]. During the outbreaks the disease causes devastation in livestock populations and economies of livestock keepers as a result of morbidity, mortality in new-borns and abortions (irrespective of gestation period) with direct negative consequences in the next crop of new-borns [9].

Public health consequences during epidemics involve a wide range of clinical manifestation in people including mild illnesses characterized by fever, muscle pain, joint pain, and headache, which can cause RVF to be confused clinically with other febrile illnesses such as malaria. In mild cases, symptoms persist for about a week and subside without specific treatment. A small percentage (0.5–2%) of patients may develop severe forms of the disease characterized by either ocular disease, meningo-encephalitis or haemorrhagic fever which last for 1–4 weeks after onset of symptoms [10, 11]. People most at risk include those in close contact with infected animals and infectious materials [4], but also those unprotected from infectious bites of infected mosquitoes. Apart from general supportive therapy, there is no established treatment for people, and a commercial vaccine for humans is not available either. The control of RVF therefore relies mainly on vaccination of livestock and preventive measures by humans (including protection from mosquito bites and avoidance of contact with infected animals and infectious material during epidemics). [11].

Inter-epidemic transmission has increasingly been reported in recent years, including in our study area, but its consequences are not fully understood and its incidence not explored enough for future epidemic preparedness [8, 12–16]. Relatively little is known regarding the natural history of RVF as the epidemics occur in remote areas inaccessible during heavy rains; on the other hand, inter-epidemic RVF transmission presents an opportunity for studying its natural history as it normally occurs when affected areas are accessible.

In Tanzania, RVF with human involvement has been reported in the past [17, 18], with few studies demonstrating inter-epidemic transmission in livestock and people [12, 19]. During the 2006/07 RVF epidemic in Tanzania, livestock and people in the Kilombero Valley were affected [20], and a sero-survey in livestock indicated presence of inter-epidemic period transmission of RVF [12]. The Kilombero Valley is a seasonally inundated floodplain between the densely forested escarpment of the Udzungwa mountains to the northwest and the grass covered Mahenge mountains to the southeast. The annual floods in the valley mimic flooding that may occur elsewhere during ENSO years. In the Kilombero Valley, there has been intense malaria transmission due to abundance of the *Anopheles gambiae* complex, but other mosquito species including vectors of RVF virus (e.g. *Culex* spp., *Aedes* spp. and *Mansonia* spp.) are present [21]. The current study therefore aimed to 1) determine whether people do acquire RVF during the inter-epidemic period in the Kilombero Valley and 2) evaluate the demographic, behavioural, occupational, and spatial determinants of recent and longstanding RVF sero-positivity in people.

## Methodology

### Study population and area

We conducted the study in rural areas of the Kilombero River Valley, located in the Kilombero and Ulanga districts in south-eastern Tanzania [22]. The Kilombero Valley is characterized by seasonal flooding which supports reproduction of large numbers of mosquitoes including arbovirus vectors such as *Aedes* spp [21]. The inhabitants of the two districts engage mainly in smallholder farming, fishing, and livestock keeping. A serological survey was carried out from March to August 2012 in six villages, three from each study district, with a total population of 14,517 in 3716 households. About a quarter of households keep livestock [23]. We selected the villages from hotspots of RVF transmission in the livestock populations in the Kilombero Valley [12]. This aimed at maximizing the probability of detecting inter-epidemic virus activity in the human population, since the hotspots indicated presence of ecological features that promote RVF transmission.

### Data and sampling

The sample size calculation took into account the fact that sampling was done in households (clusters), with an average cluster size of 5 individuals per household considered appropriate for the valley [22] so a design effect of 3 was applied. The design effect adjusted sample size was further adjusted for the expected number of covariates we hoped to evaluate, which overall gave a sample size of 726 in 145 clusters. To ensure equal representation, we selected livestock keepers’ and farmers’ households independently as sampling units, because the two sub-populations are exposed in different ways to RVF risk factors [24]. In the four villages that were within the health and demographic surveillance system (IHDSS) of the Ifakara Health Institute, we randomly selected farmers’ households from the master list of IHDSS [23]. For farmers’ households in the other two villages and for livestock keepers’ households in all villages, we obtained the lists of households from the village office and manually picked every *n* ^*th*^ household from the list.

We took blood samples from all members of the household who provided written consent to participate in the study. For children under 18 years the written consent was provided by parents or guardians. We collected blood samples into vacutainer tubes containing clot activator and after clotting, eluted the sera into cryovial tubes and kept these in a car fridge until transferred to the laboratory. We collected demographic characteristics and individuals’ exposure to risk factors to RVF through a structured questionnaire.

### Serological analyses

We analysed the serum samples for presence of RVFV antibodies by two commercial enzyme-linked immunosorbent assay (ELISA) kits, an inhibition ELISA and a capture ELISA. The inhibition ELISA simultaneously detects immunoglobulin G (IgG) and immunoglobulin M (IgM) antibodies against RVFV in humans, domestic and wildlife ruminants (Biological Diagnostic Supplies Limited, Dreghorn, United Kingdom) [25]. We converted the net optical density (OD) reading for each sample to a percentage inhibition (PI) value using the equation: [(100 –(net OD of test sample / mean net OD of negative control) x 100]. Test results producing PI values ≥38.6 are considered positive (following the manufacturer’s recommendations) whereas below that threshold is negative, with sensitivity and specificity being 99.5% and 99.7% respectively [25]. To determine recent infection, we then tested the positive samples for the presence of IgM using the capture IgM ELISA (Biological Diagnostic Supplies Limited, Dreghorn, United Kingdom) [26, 27]. For this test, we used the two intermediate net OD values of positive controls (C+) for the calculation of the net mean OD value of C+. We then used this value in subsequent calculations of percentage positivity (PP) of C+, C- and test sera as follows: [PP = (net OD serum/net mean OD C+) x 100]. The cut off for positive samples’ PP values was ≥7.1, with sensitivity and specificity being 96.4% and 99.6% respectively [27].

### Data analyses

We analysed the data in STATA version 13 (Stata Corp., College Station, Texas, USA). Samples that were positive by inhibition ELISA were considered to give evidence of past infection in the individual, as IgG antibodies last long in persons infected in the past [26]. Samples that were positive by IgM ELISA were considered to indicate recent infection in the individual, as IgM antibodies are short lived following infection by RVF virus [26, 28]. To examine risk factors of RVF virus infection and help identify households at higher risk for targeted public health interventions, we developed three separate mixed effect logistic models. We built two models for individual level risk factors for recent and past infection as outcome variables respectively and treated households as a random effect variable. We built a third model for household level factors with household sero-positivity as outcome variable and villages as random effect variable. For each model, we first determined the univariable association of individual factors with the outcome by fitting a logistic regression model. Variables with p-value <0.25 were selected as potential covariates in the multivariable analysis, where a p-value ≤ 0.05 was considered statistically significant. We performed manual forward model-building with subsequent models evaluated against sparser models by means of the Akaike Information Criterion (AIC). We also tested two-way interactions between variables included in the model. Lastly, all factors that were dropped in the process of model building were later tested for any confounding effect. We considered factors to be a confounder if they led to a change of ≥25% in the coefficient estimates. We calculated the population attributable fraction (PAF), a fraction of all cases in the study population due to exposure to a certain risk factor, as follows: PAF = (Px*(RR-1))/(1+(Px*(RR-1))), where Px = estimated population exposure and RR = risk ratio.

### Ethics statement

We obtained ethical approval from both the Institutional Review Board of the Ifakara Health Institute (IHI-IRB) and Medical Research Coordination Committee of the Tanzania’s National Institute for Medical Research for this study, permit number NIMR/HQ/R.8a/Vol.IX/1101. Prior to study procedures, participants were explained the study purpose and procedures and upon agreeing to participate, individual adult participants provided a written informed consent whereas parents or guardians provided written consent for the under-age participants.

## Results

The analyses were based on data from 606 participants in 141 households with complete questionnaire and laboratory results. We could not attain the a priori calculated sample size because of consenting issues among household members and because family size was smaller than expected. We do not anticipate this has introduced underrepresentation of participants with certain characteristics given the number of clusters involved. Out of 606 participants, 55.6% were females with age ranging between 2 and 90 years. Fifty four per cent and 46% of the participants originated from Kilombero and Ulanga districts respectively.

**Fig 1:**
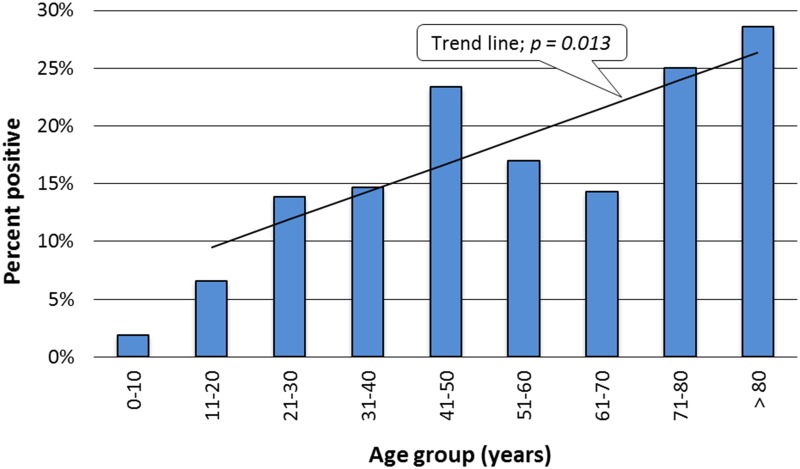
Prevalence of Rift Valley fever by age groups. The trend line indicates gradual increase of sero-positivity with age.

The inhibition ELISA results indicated an overall RVF sero-prevalence of 11.7% (95% CI = 9.2–14.5). There was a linear increase in sero-prevalence in the 10 year cohorts (Fig. 1). Evidence of recent infection by RVFV was found in 16 participants representing 2.6% overall (n = 606) and 22.5% of inhibition ELISA positive individuals (n = 71). Four of 16 (25.0%) IgM positives and 11/71 (15.5%) of individuals with inhibition ELISA sero-positivity reported they had had no previous animal contact, suggesting that at least part of the transmission in the area occurred through infected mosquito bites.

In the univariable analyses, factors associated with past RVF infection were history of participating in slaughter of animals (odds ratio [OR] 1.85; 95% CI 1.01–3.42), assisting birthing animals (OR 2.02; 95% CI 1.12–3.63), milking animals (OR 2.45; 95% CI 1.35–4.45), eating raw meat/blood (OR 6.01; 95% CI 1.86–19.39), disposing aborted foetus (OR 2.04; 95% CI 1.13–3.67) and being older (OR 1.03 per year; 95% CI 1.02–1.04) (Table 1). In the multivariable model, age (OR 1.03; 95% CI 1.01–1.04), milking animals (OR 2.19; 95% CI 1.23–3.91) and eating raw meat/blood (OR 4.17; 95% CI 1.18–14.66) remained significantly associated with past infection (Table 2). The PAFs of milking animals and eating raw meat in the past were 29% and 6% respectively. None of the risk factors studied were associated with recent infection (results not shown).

**Table 1:** Prevalence of RVF inhibition ELISA sero-positivity and association of individual-level variables with sero-positivity.

S/No.	Factor	Level	%Positive (n)	OR	95% CI
1	District	Kilombero	11.6 (327)	1	
		Ulanga	11.8 (279)	1.01	0.53–1.79
2	Village	Iragua	12.6 (119)	1	
		Lungongole	10.2 (137)	0.75	0.31–1.84
		Lupiro	12.0 (75)	0.91	0.33–2.48
		Mofu	15.8 (101)	1.33	0.55–3.21
		Nakafuru	10.5 (85)	0.84	0.31–2.25
		Sagamaganga	8.9 (89)	0.70	0.25–1.93
3	Sex	Female	10.6 (337)	1	
		Male	13.0 (269)	1.26	0.75–2.12
4	** Age (year categories)	0–10	1.9 (105)	1	
		11–20	6.5 (168)	3.59	0.76–16.83
		21–30	13.8 (108)	9.02	1.94–41.90
		31–40	14.7 (68)	8.95	1.85–43.31
		41–50	23.3 (77)	16.97	3.67–78.43
		51–60	17.0 (47)	10.87	2.12–55.62
		61–70	14.2 (14)	10.24	1.21–86.54
		71–80	25.0 (12)	19.71	2.65–146.67
		> 80	28.5 (7)	21.43	2.22–206.86
5	Occupation	Farmer	11.9 (242)	0.119	0.081–0.167
		Livestock keeper	11.7 (356)	0.117	0.086–0.156
		Other	0.0 (8)	0.000	0.000–0.369
6	Bed net ownership	Yes	11.4 (577)	0.56	0.18–1.73
		No	19.2 (26)	1	
7	Bed net use	Yes	11.0 (532)	0.75	0.34–1.63
		No	16.4 (73)	1	
8	Keeping livestock	Yes	12.0 (365)	1.10	0.61–1.97
		No	11.2 (241)	1	
9	** Slaughter animal in the past	Yes	17.2 (110)	1.85	1.01–3.42
		No	10.5 (493)	1	
10	Slaughter a sick animal in the past	Yes	16.6 (30)	1.47	0.56–3.88
		No	11.5 (562)	1	
11	** Eat raw meat	Yes	42.8 (14)	6.01	1.86–19.39
		No	10.9 (583)	1	
12	* Eat meat from dead animal	Yes	14.0 (249)	1.59	0.89–2.85
		No	9.8 (314)	1	
		Don’t know	14.2 (35)	1.50	0.51–4.46
13	** Milking	Yes	16.5 (254)	2.45	1.35–4.45
		No	8.2 (350)	1	
14	Drink raw milk	Yes	12.2 (450)	1.28	0.67–2.43
		No	9.8 (152)	1	
15	** Help with birthing animal	Yes	17.3 (127)	2.02	1.12–3.63
		No	9.6 (458)	1	
16	** Dispose of aborted foetus	Yes	18.5 (113)	2.04	1.13–3.67
		No	10.1 (464)	1	

Significance levels at univariable mixed effect logistic regression model,

** < 0.05;

* > 0.05 but < 0.25

**Table 2:** Multivariable analysis of correlates of RVF antibody sero-positivity.

No.	Factor	Level	OR	95% CI
**1**	Help with birthing animal	No	1	
		Yes	0.83	0.36–1.90
**2**	Age (years)	n/a*	1.03	1.01–1.04
**3**	Milking	No	1	
		Yes	2.19	1.23–3.91
**4**	Eat raw meat	No	1	
		Yes	4.17	1.18–14.66
**5**	Dispose of aborted foetus	No	1	
		Yes	1.35	0.59–3.09

* Age was included as a continuous variable,

OR = odds ratio,

CI = confidence interval

Though keeping livestock was not associated with individuals’ sero-positivity, households keeping livestock had a higher chance of having at least one member with past infection (OR = 3.04, 95% CI = 1.42–6.48) than households that do not keep livestock (table 3). Participant’s gender, eating meat from dead animals, drinking raw milk, bed net use, proximity to the main flood area, elevation and district were not associated with inhibition ELISA sero-positivity.

**Table 3:** Household-level factors for RVF sero-positivity.

No.	Factor	Level	OR	95% CI
**1**	Keep livestock	No	1	
		Yes	3.04	1.42–6.48
**2**	Elevation (meters)	n/a*	0.98	0.97–1.00

* Elevation was included as a continuous variable,

OR = odds ratio,

CI = confidence interval

## Discussion

We report here presence of IgM antibodies against RVFV among inhabitants of Kilombero Valley. This confirms recent infection and thus transmission of RVF which is not linked to the previous epidemic which happened five years prior in the study area [20]. This finding affirms our previous report, which highlighted IEP transmission of RVF in livestock [12]. Inter-epidemic sero-positivity to RVF in people has also been previously documented in other parts of Tanzania and Africa [13, 14, 16, 29], with IgM antibodies detected in Nigeria and Chad [14, 29]. The observed sero-prevalence by inhibition ELISA (11.7%) in this study is high compared to studies from other parts of Tanzania during inter-epidemic period with sero-prevalence of 5.2% and 4% in Mbeya and Tanga regions respectively [13, 19], possibly as a result of our sampling of participants from hotspots of RVF circulation in animals. In Gabon, a country with no RVF epidemic history, a sero-prevalence of 3.3% has been reported [30], in Kenya, an epidemic prone country, a mixed picture for inter-epidemic sero-positivity has been recorded in people in different geographical locality and time [16, 31].

In this study, participants who milked animals were more likely to have evidence of past RVF infection. This points to a potential public health consequence of RVFV shedding in milk which occurs during the viraemic phase of the disease. The traditional milking practices create a lot of aerosols, and if one is milking a viraemic animal the RVFV containing milk particles could result into infection to milkers through inhalation of the infectious aerosols [32]. Also skin abrasions on hands of milkers could form an easy route of infection when people have broken skin. However, drinking raw milk was not associated with longstanding sero-positivity. Although raw milk consumption is considered an important risk factor during epidemics [18, 33], the infection through oral route comes across barriers including acidic environment in the stomach [34]. The findings in our study might also be explained by the practice of consuming fermented milk by the livestock keepers in which case the virus would die when exposed to acidic environment of sour milk [34].

People who ate raw meat (including blood and internal organs such as kidneys and liver) were more likely to have evidence of past RVF infection. The animal products (meat and blood) from infected animals could have a high concentration of RVFV which has the ability to persist at neutral pH in carcasses. When meat is consumed raw before the pH drops with rigor mortis this could lead to infection in people. Eating meat from animals that died before slaughter was not associated with sero-positivity which might be because individuals who reported eating meat from dead animals also reported cooking the meat before consumption, which would have destroyed the virus.

The high PAF values for milking and for eating raw meat as risk factors present important educational intervention targets for risk reduction even during epidemic free periods. The increased sero-prevalence in older individuals suggests stable rates of on-going transmission in the population. The increased sero-prevalence was also evident when participants were categorized into ten-year cohorts, with drops in the 51–60 and 61–70 groups. Older individuals might have either been infected in one or more previous epidemics or through clinically undetected low-level virus circulation in the study area.

Although there was no significant risk difference between individual livestock keepers and farmers, households keeping livestock had a higher probability of having at least one member with past RVF infection. This might imply presence of either higher risk through animal contact as compared to mosquito bites or higher exposure to infectious mosquito bites among livestock keepers, as mosquitoes living in close proximity to livestock can pick up infection from amplifying infected hosts and transmit to livestock keepers even in circumstances of low-level virus circulation in the general vector populations.

Helping with birthing animals and disposal of aborted foetuses are high risk activities when dealing with infected animals or infectious materials especially when not wearing proper protective attire. In this study both were not statistically significant in the final model. People who reported participating in slaughtering animals in the past (including skinning and butchering) were more likely to be sero-positive but the sero-positivity was not associated with slaughtering sick animals suffering from other unknown conditions. Slaughtering animals sick from RVF exposes individuals through direct contact with infectious materials such as aerosols from oozing blood and other organs during skinning and butchering [3].

Although sero-prevalence in male individuals was slightly higher, sex was not associated with sero-positivity. The sex difference in RVF prevalence has been reported in some studies [16, 30] but was not apparent in others [13, 19, 35] and where it existed, it has been mostly attributed to gender-biased distribution of animal handling in affected populations. The lack of association between gender and sero-positivity in this study indicates that either the specific risk-increasing animal handling activities are equally distributed between genders or that direct mosquito bites as source of infection to people in the valley was equally important. The latter possibility is supported in our study area because men were more involved with animal handling duties.

Bed net ownership and use were not associated with sero-positivity. This is possibly because in the study area there is high bed net coverage [36], but also because the main RVF vector *Aedes* mosquitoes are day biting mosquitoes.

### Conclusion

These findings, coupled with our previous report in livestock [12], indicate persistent IEP transmission of RVFV in both livestock and human populations in the Kilombero Valley. The animal contact risk factors, especially milking and eating raw meat are important and present educational intervention targets for risk reduction. In the wake of declining malaria incidence [37] these findings underscore the need for clinicians to consider RVF in the differential diagnosis for febrile illnesses among Kilombero Valley inhabitants. This is relevant regardless of the person’s occupation, because part of the transmission likely happens through infectious mosquito bites. The findings also suggest the opportunity and need to further investigate the circulating RVFV strain as well as the main vectors responsible for IEP transmission.

## Supporting Information

S1 Checklist STROBE Checklist. (DOC)

## References

[1] Bird BH, Ksiazek TG, Nichol ST and MacLachlan NJ (2009) Rift Valley Fever Virus. J Am Vet Med Assoc. 234(7): 883–893. 10.2460/javma.234.7.883 19335238

[2] Swanepoel R and Coetzer JAW, Rift Valley Fever, in Infectious Diseases of Livestock in Southern Africa, Coetzer J.A.W. and Tustin R.C., Editors. 2004, Oxford University Press p. 1037–1059.

[3] Abu-Elyazeed R, El-Sharkawy S, Olson J, Botros B, Soliman A, et al (1996) Prevalence of Anti-Rift-Valley-Fever Igm Antibody in Abattoir Workers in the Nile Delta During the 1993 Outbreak in Egypt. Bull World Health Organ. 74(2): 155 8706230 PMC2486897

[4] Archer BN, Weyer J, Paweska J, Nkosi D, Leman P, et al (2011) Outbreak of Rift Valley Fever Affecting Veterinarians and Farmers in South Africa, 2008. S Afr Med J. 101(4): 263–266. 21786732 10.7196/samj.4544

[5] McIntosh B, Russell D, Dos Santos I and Gear J (1980) Rift Valley Fever in Humans in South Africa. S Afr Med J. 58(20): 803–806. 7192434

[6] Daubney R, Hudson JR and Garnham PC (1931) Enzootic Hepatitis or Rift Valley Fever: An Undescribed Virus Disease of Sheep, Cattle and Man from East Africa. J Pathol. 34(4): 545–579.

[7] Anyamba A, Chretien J-P, Small J, Tucker CJ, Formenty PB, et al (2009) Prediction of a Rift Valley Fever Outbreak. Proc Natl Acad Sci USA. 106(3): 955–959. 10.1073/pnas.0806490106 19144928 PMC2626607

[8] Chevalier V, Thiongane Y and Lancelot R (2009) Endemic Transmission of Rift Valley Fever in Senegal. Transbound Emerg Dis. 56: 372–374. 10.1111/j.1865-1682.2009.01083.x 19548898

[9] Rich KM and Wanyoike F (2010) An Assessment of the Regional and National Socio-Economic Impacts of the 2007 Rift Valley Fever Outbreak in Kenya. Am J Trop Med Hyg. 83(2 Suppl): 52–57. 10.4269/ajtmh.2010.09-0291 20682906 PMC2913501

[10] Madani TA, Al-Mazrou YY, Al-Jeffri MH, Mishkhas AA, Al-Rabeah AM, et al (2003) Rift Valley Fever Epidemic in Saudi Arabia: Epidemiological, Clinical, and Laboratory Characteristics. Clin Infect Dis. 37(8): 1084–1092. 14523773 10.1086/378747

[11] WHO (2008) Rift Valley Fever Fact Sheet. Wkly Epidemiol Rec. 83(2): 17–24. 18188879

[12] Sumaye RD, Geubbels E, Mbeyela E and Berkvens D (2013) Inter-Epidemic Transmission of Rift Valley Fever in Livestock in the Kilombero River Valley, Tanzania: A Cross-Sectional Survey. PLoS Negl Trop Dis. 7(8): e2356 10.1371/journal.pntd.0002356 23951376 PMC3738442

[13] Swai ES and Schoonman L (2008) Prevalence of Rift Valley Fever Immunoglobulin G Antibody in Various Occupational Groups before the 2007 Outbreak in Tanzania. Vector Borne Zoonotic Dis. 0(0): 1–4. 10.1089/vbz.2008.0108 19125662

[14] Durand JP, Bouloy M, Richecoeur L, Peyrefitte CN and Tolou H (2003) Rift Valley Fever Virus Infection among French Troops in Chad. Emerg Infect Dis. 9(6): 751–752. 12781023 10.3201/eid0906.020647 PMC3000143

[15] LaBeaud AD, Cross PC, Getz WM, Glinka A and King CH (2011) Rift Valley Fever Virus Infection in African Buffalo (Syncerus Caffer) Herds in Rural South Africa: Evidence of Interepidemic Transmission. Am J Trop Med Hyg. 84(4): 641–646. 10.4269/ajtmh.2011.10-0187 21460024 PMC3062463

[16] LaBeaud AD, Muchiri EM, Ndzovu M, Mwanje MT, Muiruri S, et al (2008) Interepidemic Rift Valley Fever Virus Seropositivity, Northeastern Kenya. Emerg Infect Dis. 14(8): 1240 10.3201/eid1408.080082 18680647 PMC2600406

[17] Mohamed M, Mosha F, Mghamba J, Zaki SR, Shieh WJ, et al (2010) Epidemiologic and Clinical Aspects of a Rift Valley Fever Outbreak in Humans in Tanzania, 2007. Am J Trop Med Hyg. 83(Suppl 2): 22–27. 20682902 10.4269/ajtmh.2010.09-0318 PMC2913502

[18] Woods CW, Karpati AM, Grein T, McCarthy N, Gaturuku P, et al (2002) An Outbreak of Rift Valley Fever in Northeastern Kenya, 1997–98. Emerg Infect Dis. 8(2): 138–144. 11897064 10.3201/eid0802.010023 PMC2732454

[19] Heinrich N, Saathoff E, Weller N, Clowes P, Kroidl I, et al (2012) High Seroprevalence of Rift Valley Fever and Evidence for Endemic Circulation in Mbeya Region, Tanzania, in a Cross-Sectional Study. PLoS Negl Trop Dis. 6(3): e1557 10.1371/journal.pntd.0001557 22479657 PMC3313937

[20] WHO (2007) Outbreaks of Rift Valley Fever in Kenya, Somalia and United Republic of Tanzania, December 2006–April 2007. Wkly Epidemiol Rec. 82: 169–178. 17508438

[21] Ogoma SB, Lweitoijera DW, Ngonyani H, Furer B, Russell TL, et al (2010) Screening Mosquito House Entry Points as a Potential Method for Integrated Control of Endophagic Filariasis, Arbovirus and Malaria Vectors. PLoS Negl Trop Dis. 4(8): e773 10.1371/journal.pntd.0000773 20689815 PMC2914752

[22] Population and Household Census General Report, 2013: Dar es Salaam. p. 264.

[23] Armstrong-Schellenberg J, Mukasa O, Abdulla S, Marchant T, Lengeler C, et al, Chapter 11 Ifakara Dss, Tanzania, in Population and Health in Developing Countries: Volume 1 Population, Health, and Survival in Indepth Sites. 2002, International Development Research Centre: Ottawa p. 159–164.

[24] Anyangu AS, Gould LH, Sharif SK, Nguku PM, Omolo JO, et al (2010) Risk Factors for Severe Rift Valley Fever Infection in Kenya, 2007. Am J Trop Med Hyg. 83(2_Suppl): 14 10.4269/ajtmh.2010.09-0293 20682901 PMC2913492

[25] Paweska JT, Mortimer E, Leman PA and Swanepoel R (2005) An Inhibition Enzyme-Linked Immunosorbent Assay for the Detection of Antibody to Rift Valley Fever Virus in Humans, Domestic and Wild Ruminants. J Virol Methods. 127(1): 10–18. 15893560 10.1016/j.jviromet.2005.02.008

[26] Paweska JT, Burt FJ, Anthony F, Smith SJ, Grobbelaar AA, et al (2003) Igg-Sandwich and Igm-Capture Enzyme-Linked Immunosorbent Assay for the Detection of Antibody to Rift Valley Fever Virus in Domestic Ruminants. J Virol Methods. 113(2): 103–112. 14553896 10.1016/s0166-0934(03)00228-3

[27] Paweska JT, Burt FJ and Swanepoel R (2005) Validation of Igg-Sandwich and Igm-Capture Elisa for the Detection of Antibody to Rift Valley Fever Virus in Humans. J Virol Methods. 124(1): 173–181. 15664066 10.1016/j.jviromet.2004.11.020

[28] Morvan J, Rollin PE, Laventure S and Roux J (1992) Duration of Immunoglobulin M Antibodies against Rift Valley Fever Virus in Cattle after Natural Infection. Trans R Soc Trop Med Hyg. 86(6): 675–675. 1287943 10.1016/0035-9203(92)90187-h

[29] Olaleye OD, Tomori O, Ladipo MA and Schmitz H (1996) Rift Valley Fever in Nigeria: Infections in Humans. Rev Sci Tech. 15(3): 923–935. 9025142 10.20506/rst.15.3.967

[30] Pourrut X, Nkoghe D, Souris M, Paupy C, Paweska J, et al (2010) Rift Valley Fever Virus Seroprevalence in Human Rural Populations of Gabon. PLoS Negl Trop Dis. 4(7): e763 10.1371/journal.pntd.0000763 20668541 PMC2910672

[31] Labeaud AD, Ochiai Y, Peters CJ, Muchiri EM and King CH (2007) Spectrum of Rift Valley Fever Virus Transmission in Kenya: Insights from Three Distinct Regions. Am J Trop Med Hyg. 76(5): 795–800. 17488893 PMC2367216

[32] Morrill JC and Peters C (2011) Mucosal Immunization of Rhesus Macaques with Rift Valley Fever Mp-12 Vaccine. J Infect Dis. 204(4): 617–625. 10.1093/infdis/jir354 21791664

[33] LaBeaud AD, Muiruri S, Sutherland LJ, Dahir S, Gildengorin G, et al (2011) Postepidemic Analysis of Rift Valley Fever Virus Transmission in Northeastern Kenya: A Village Cohort Study. PLoS Negl Trop Dis. 5(8): e1265 10.1371/journal.pntd.0001265 21858236 PMC3156691

[34] Easterday BC (1965) Rift Valley Fever. Adv Vet Sci. 10: 65–127. 5331042

[35] Wilson ML, Chapman LE, Hall DB, Dykstra EA, Ba K, et al (1994) Rift Valley Fever in Rural Northern Senegal: Human Risk Factors and Potential Vectors. Am J Trop Med Hyg. 50(6): 663–675. 7912905 10.4269/ajtmh.1994.50.663

[36] Russell TL, Lwetoijera DW, Maliti D, Chipwaza B, Charlwood JD, et al (2010) Impact of Promoting Longer-Lasting Insecticide Treatment of Bed Nets Upon Malaria Transmission in a Rural Tanzanian Setting with Pre-Existing High Coverage of Untreated Nets. Malar J. 9(187). 10.1186/1475-2875-9-187 20579399 PMC2902500

[37] WHO, World Malaria Report. 2013: World Health Organization.

